# Socioeconomic differences in one-year survival after ischemic stroke: the effect of acute and post-acute care-pathways in a cohort study

**DOI:** 10.1186/s12889-016-3019-8

**Published:** 2016-05-16

**Authors:** Valeria Belleudi, Paolo Sciattella, Nera Agabiti, Mirko Di Martino, Riccardo Di Domenicantonio, Marina Davoli, Danilo Fusco

**Affiliations:** Department of Epidemiology, Lazio Regional Health Service, Via Cristoforo Colombo, 112, 00147 Rome, Italy

**Keywords:** Socioeconomic-position, Stroke, Pathway-analysis, Survival

## Abstract

**Background:**

The reasons for socioeconomic inequity in stroke mortality are not well understood. The aim of this study was to explore the role of ischemic stroke care-pathways on the association between education level and one-year survival after hospital admission.

**Methods:**

Hospitalizations for ischemic stroke during 2011/12 were selected from Lazio health data. Patients’ clinical history was defined by retrieving previous hospitalizations and drugs prescriptions. The association between education level and mortality after stroke was studied for acute and post-acute phases using multilevel logistic models (Odds Ratio (OR)). Different scenarios of quality care-pathways were identified considering hospital performance, access to rehabilitation and drug treatment post-discharge. The probability to survive to acute and post-acute phases according to education level and care-pathway scenarios was estimated for a “mean-severity” patient. One-year survival probability was calculated as the product of two probabilities. For each scenario, the 1-year survival probability ratio, university versus elementary education, and its Bootstrap Confidence Intervals (95 % BCI) were calculated.

**Results:**

We identified 9,958 patients with ischemic stroke, 53.3 % with elementary education level and 3.2 % with university. The mortality was 14.9 % in acute phase and 14.3 % in post-acute phase among survived to the acute phase. The adjusted mortality in acute and post-acute phases decreased with an increase in educational level (OR = 0.90 *p*-trend < 0.001; OR = 0.85 *p*-trend < 0.001). For the best care-pathway, the one-year survival probability ratio was 1.06 (95 % BCI = 1.03–1.10), while it was 1.17 (95 % BCI = 1.09–1.25) for the worst.

**Conclusions:**

Education level was inversely associated with mortality both in acute and post-acute phases. The care-pathway reduces but does not eliminate 1-year survival inequity.

## Background

The relationship between risk of dying from a stroke and Socio-Economic Status (SES), identified by income or education level, has been extensively investigated [1–3]. Most of the studies have shown an inverse association between stoke incidence/mortality and SES [4]. However, the mechanisms by which SES affects stroke mortality have not been established. The social gradient observed may be associated with worse lifestyles/clinical conditions that are typically observed in groups of people with a relatively low socioeconomic position; [5, 6] however, several studies have also suggested a potential association between SES and quality of care [7–10]. Evidence exists demonstrating that early management in a specialist environment for acute stroke patients is associated with health outcomes; however, conflicting results exist regarding the beneficial effects of pharmacological treatments in acute and post-acute phases [11]. Early and post-acute rehabilitation is clearly recommended, and optimal strategies for patients with various levels of neurological impairment after stroke need to be elucidated [11]. The limits of randomized controlled trial and the need for observational research to evaluate the real-life effectiveness of health interventions have recently been strongly emphasized [12].

The aims of this study were as follows: to analyze the effect of SES on short- and long-term mortality after acute ischemic stroke; to measure the real-life effectiveness of the acute and post-acute pathways of care for patients with acute ischemic stroke; and to explore the role of the care-pathway, both for the acute and post-acute phases, on the association between education level and one-year survival after admission for ischemic stroke.

## Methods

### Data sources

A health data repository for the Lazio region in Italy (approximately 5 million residents) is available. It connects health information for each individual registered in the Regional Health System (approximately 97 % of residents) using a unique and anonymous subject identifier. Specifically, the available health information present in this system includes hospitalizations, emergency visits, rehabilitation, drug prescriptions and mortality.

### Study population

From the Lazio health data warehouse, we identified a cohort of patients, all aged over 35 years old, discharged from the hospital with a diagnosis of ischemic stroke between January 1st 2011 and December 31st 2012 (ICD-9-CM codes: 433.x1, 434.×1, 436) and survivors to at least 1 day post-admission. We excluded patients with a previous hospitalization for stroke, either hemorrhagic or ischemic, in the 2 years previous to admission.

### Patient characteristics

For each subject, the socio-demographic factors, including age, sex and educational level, recorded during the ischemic stroke hospitalization were considered. Patient clinical history was defined by retrieving specific conditions recorded during hospitalizations or emergency visits in the 2 years previous to the index stroke admission and by taking into account concomitant conditions with the index admission. The conditions retrieved were diabetes, chronic obstructive pulmonary disease, hypertension, previous myocardial infarction, heart failure, rheumatic heart disease, cardiomyopathy acute endocarditis and myocarditis, other heart conditions, conduction disturbances and arrhythmias, cerebrovascular diseases, vascular diseases, obesity-dyslipidemia, blood disorders, chronic digestive disease, chronic renal diseases, and cancer.

To better define the clinical profiles of the patients, we also assessed the use of drugs in the 6 months prior to admission: cardiac therapies, anti-diabetic drugs, antiplatelet therapies, anticoagulants, antihypertensive drugs, diuretics, beta-blocking agents, calcium channel blockers, angiotensin-converting-enzyme inhibitors, and angiotensin II antagonists or statins.

### Outcomes

Two different post-stroke outcomes were defined: 2–30 days mortality for the acute-phase and 31–365 days mortality for the post-acute phase.

### Care-pathways

To identify different scenarios of quality care-pathways in the acute phase, we classified hospital performance considering the available literature. Hospitals with a stroke unit or a team of expert neurologists were defined as “high performance” [9, 13–15], while the hospitals with a low volume of stroke admissions (<100 in the study period) were defined as “low performance” [7, 16]. All others were classified as “medium performance”. The care-pathway during the post-acute phase was defined on the basis of access to rehabilitation or drug treatment in the 30 days after discharge: number of antihypertensive, antithrombotic, or statin drugs. To reduce misclassification and heterogeneity of care-pathways in the post-acute phase, we excluded patients who died within 30 days after discharge or with a length of stay in the hospital ≥ 28 days. We defined the best care-pathway scenario as a “hospital with high performance, access to rehabilitation, use of all three drugs” and the worst as a “hospital with low performance, no access to rehabilitation, no use of drugs”.

### Statistical analysis

The association between education level and mortality after stroke was studied for both the acute and post-acute phases using logistic models [Odds Ratio (OR)] and adjusting for age, sex, care-pathway and the risk factors selected by a stepwise bootstrap procedure. Using this approach, 1000 replicated bootstrap samples were selected from the original cohort. A bootstrap sample is a sample of the same size as the original dataset chosen with replacement. Thus, a given subject in the original cohort may be selected multiple times, only once, or not at all in a specific bootstrap sample. A stepwise procedure, using thresholds of *p* = 0.05 for variable selection and elimination, was applied to each replicated sample, and only the factors selected in at least 50 % of the procedures were included in the final models. Taking into account the variability of mortality between hospitals, we used a multilevel approach, with hospital as 2nd level, considering patients as repeated observations within hospitals.

To evaluate the effect of increase of education level on mortality, we estimate OR considering exposure as a linear variable.

The probability to survive to the acute and post-acute phases according to education level and care-pathway scenarios were estimated for a “mean severity” patient, assuming for this patient the same distribution of age, sex and mortality risk factors as observed in the cohort. Because the survival of patients in the post-acute phase (B) is conditioned to the survival in the acute phase (A), the 1-year survival probability was calculated as the product of the two probabilities: P(1-year survival) = P(A)P(B|A).

For each scenario, the 1-year probability ratio for university versus elementary education and its Bootstrap Confidence Intervals (95 % BCI) were calculated. In this sense, the bootstrap distribution represents an (approximate) nonparametric, noninformative posterior distribution for our parameter. But this bootstrap distribution is obtained painlessly, without having to formally specify a prior and without having to sample from the posterior distribution. By perturbing the data, the bootstrap approximates the Bayesian effect of perturbing the parameters, and is typically much simpler to carry [17].

The 1-year Probability Ratios (PRs) for the best and worst scenarios were calculated by sex and age classes to evaluate the potential effect of demographic factors on the relationship among care-pathways, education level and mortality.

### Sensitivity analyses

To assess the robustness of the results, three sensitivity analyses were carried out. To take into account the effect of the time spent in the post-acute phase, the association between education level and mortality was examined using an adjusted Cox model [Hazard Ratio (HR)]. To confirm the strength of our findings, we replied the main analysis on the subgroup of new drug users. To reduce the selection bias due to the exclusion of patients in the post-acute phase, we applied the main analysis using the Inverse Probability Weighting (IPW). This methodology allows to correct the analysis by weighting the observations with the probability of being selected. The IPW is based on the assumption that individual information that can predict the probability of inclusion (non-missingness) are available for the entire study population, so that, after taking account of them, we can make inferences about the entire target population starting from the non-missing observations alone. The procedure for the calculation was the following: firstly, we considered the entire population at study and calculated the probability of non-missing information using a logistic regression model, where the response was the non-missingness and the covariates are its possible predictors. The weight of each subject was given by the inverse of the predicted probability. Then the analysis was performed only on the non-missing observations using a weighted model.

## Results

**Fig. 1:**
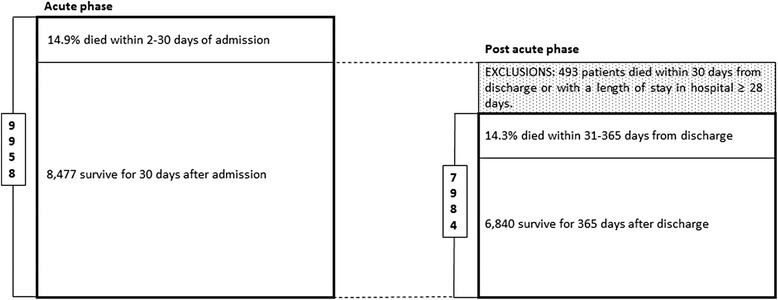
Number of patients and mortality in the acute and post-acute phases

We identified 9,958 patients who were hospitalized for ischemic stroke during 2011–2012 (Fig. 1). Among them, 8,477 (85.1 %) survived for 30 days after admission, and 5.8 % were excluded from the post-acute phase. The overall 1-year mortality was 26.3 %.

The mean age of the cohort was 76 years, 50 % were male, 53.3 % had an elementary education level, and 3.2 % had a university degree (Table 1). The adjusted mortality in the acute and post-acute phases decreased with an increase in the education level (OR = 0.90 p-trend < 0.001; OR = 0.85 p-trend < 0.001). In particular, we observed a no statistically significant reduction in mortality of 31 % for patients with a university-level education relative to those with an elementary-level education in the acute phase (OR 0.69; *p*.value = 0.096) and of 45 %, statistically significant, in the post-acute phase (OR 0.55; *p*.value = 0.021). Hospitals with high performance had lower mortality in the acute phase (OR = 0.70; *p*.value = 0.006); this protective effect also persisted in the post-acute phase, but it was not significant (OR = 0.82; *p*.value = 0.106).

**Table 1:** Determinants of mortality after stroke for the acute and post-acute phases

	Acute phase 2–30 days (*N* = 9958)			Post acute phase 31–365 days (*N* = 7984)		
	% of patients	OR_adj_^a^	*p* value	% of patients	OR_adj_^a^	*p* value
*Sex*						
Women vs Men	50.0	1.18	0.010	47.3	1.02	0.824
*Age in years*						
35–65	18.1	1.00	<.0001^c^	21.6	1.00	<.0001^c^
66–75	23.2	2.45	<.0001	25.9	2.24	<.0001
76–85	38.2	5.20	<.0001	37.7	3.75	<.0001
85+	20.4	11.17	<.0001	14.8	7.96	<.0001
*Education level*						
Elementary	53.3	1.00	0.038^c^	49.5	1.00	0.101^c^
Medium	28.7	0.88	0.132	30.7	0.87	0.123
High	14.8	0.71	0.004	16.4	0.81	0.092
University	3.2	0.69	0.096	3.4	0.55	0.021
*Care-Pathway*						
Hospital Performance						
Medium	38.5	1.00	0.025^c^	37.6	1.00	0.523^c^
High	41.6	0.70	0.006	43.9	0.82	0.106
Low	19.9	1.21	0.156	18.6	0.99	0.917
Access to rehabilitation				33.0	0.93	0.402
Treatment post-discharge^b^						
Three drugs				23.0	1.00	<.0001^c^
Two drugs				27.7	1.58	<.0001
One drugs				15.5	2.15	<.0001
No drugs				33.7	3.43	<.0001
*p*-value (test di Wald)	0.014			0.029		

^a^adjusted for risk factors selected from clinical history and previous use of drugs

^b^number of antihypertensive, antithrombotic, or statin drugs

^c^global Chi-Square test

Access to rehabilitation post-discharge did not change the long-term mortality (OR = 0.93; *p*.value = 0.402). For each drug that was subtracted from “complete” drug treatment, we observed an increase in mortality in the post-acute phase (OR = 1.49; p-trend < 0.001; data not shown).

**Fig. 2:**
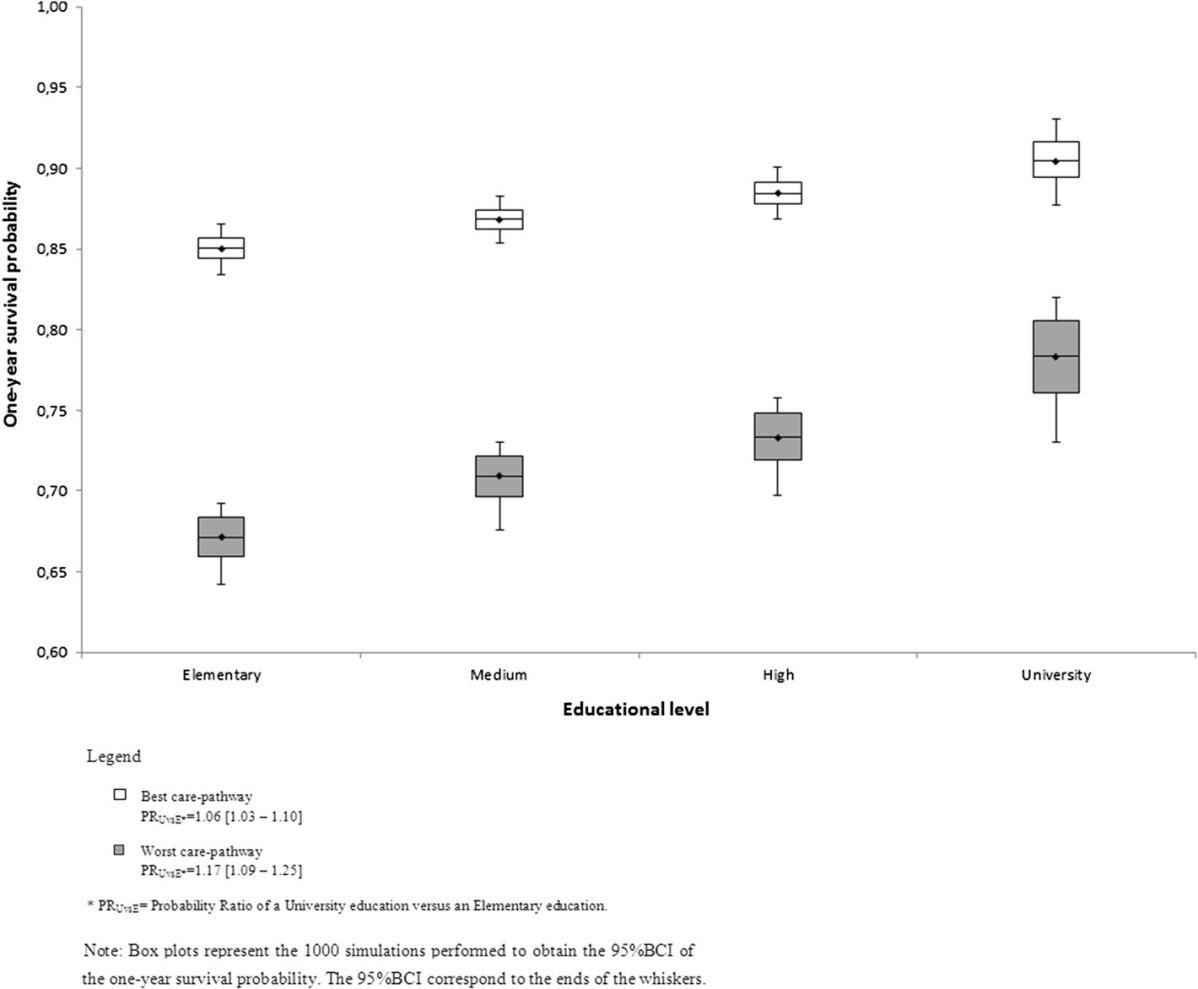
One-year survival probability for education level according to best and worst care-pathways for a “mean severity” patient. □ Best care-pathway. PR_UvsE*_ = 1.06 [1.03 – 1.10].  Worst care-pathway. PR_UvsE*_ = 1.17 [1.09 – 1.25]. * PR_UvsE_ = Probability Ratio of a University education versus an Elementary education. Note: Box plots represent the 1000 simulations performed to obtain the 95 % BCI of the 1 one-year survival probability. The 95 % BCI correspond to the ends of the whiskers

Figure 2 shows the 1-year probability to survive by education level according to the best and worst care-pathway for a “mean severity” patient. For the best care-pathway, the 1-year probability ratio of a university education versus an elementary education was 1.06 (95 % BCI = 1.03–1.10), whereas it was 1.17 (95 % BCI = 1.09–1.25) for the worst care-pathway.

**Fig. 3:**
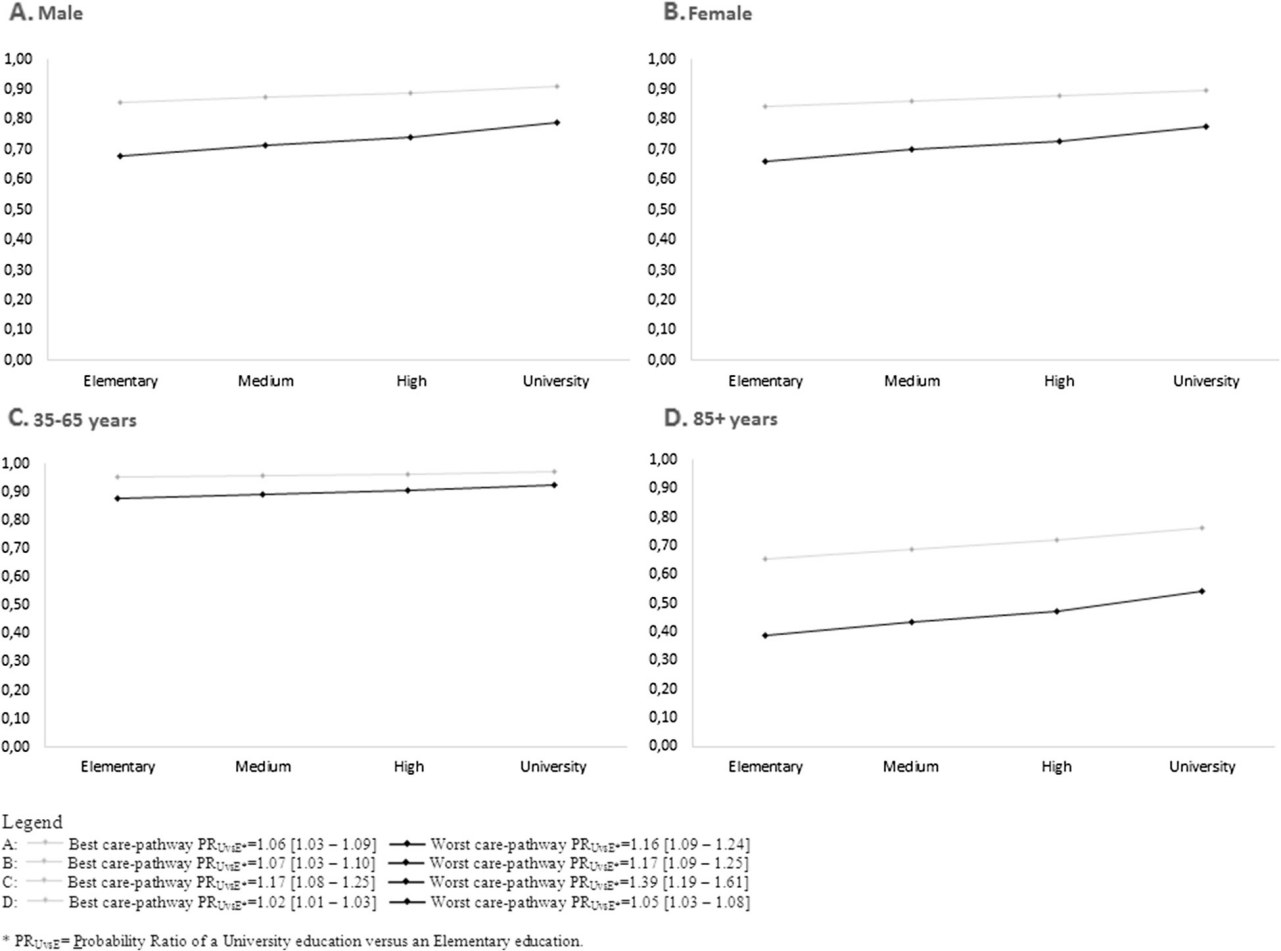
One-year survival probability by education level according to the best and worst care-pathway for men, women, younger (35–65) and older (85+) patients. **a**:  Best care-pathway PR_UvsE*_ = 1.06 [1.03 – 1.09]  Worst care-pathway PR_UvsE*_ = 1.16 [1.09 – 1.24]. **b**:  Best care-pathway PR_UvsE*_ = 1.07 [1.03 – 1.10]  Worst care-pathway PR_UvsE*_ = 1.17 [1.09 – 1.25]. **c**:  Best care-pathway PR_UvsE*_ = 1.17 [1.08 – 1.25]  Worst care-pathway PR_UvsE*_ = 1.39 [1.19 – 1.61]. **d**:  Best care-pathway PR_UvsE*_ = 1.02 [1.01 – 1.03]  Worst care-pathway PR_UvsE*_ = 1.05 [1.03 – 1.08]. * PR_UvsE_ = Probability Ratio of a University education versus an Elementary education

The relationships among care-pathway, education level and 1-year survival were investigated for demographic characteristics (Fig. 3). The relationship did not change between men and women; for younger patients, the effect of the care-pathway on the association between education level and 1-year probability of survival was reduced (best care-pathway: PR = 1.02 vs 1.06; worst care-pathway: PR = 1.05 vs 1.17). However, for older patients, the effect was accentuated (best care-pathway: PR = 1.17 vs 1.06; worst care-pathway: PR = 1.39 vs 1.17).

The three sensitivity analyses confirmed the main results. In particular, in the post-acute phase, the adjusted risk of mortality for patients with a university education in respect to those with an elementary education was as follows: HR = 0.55 with a *p*-value of 0.012 using the Cox model and OR = 0.40 with a *p*-value of 0.010 applying the analysis for new users only. Finally, when we applied inverse probability weighting, we obtained that the patients excluded in post-acute phase were older, with a higher presence of comorbidities, such as diabetes and heart failure, and a greater use of diuretics. In this sub-analysis the 1-year probability ratio university versus elementary education was 1.03 for the best care-pathway and 1.11 for the worst care-pathway.

## Discussion

We found a negative association between education level and mortality after hospital admission for ischemic stroke both in the acute and post-acute phases. Patients who experienced the best care-pathway, in terms of a high-performance hospital and access to rehabilitation and drug treatment, had a higher 1-year survival. Among the patients who were treated with the best care-pathway, the socioeconomic associated differential in mortality was lower than among those patients who received the worst care-pathway.

The inverse association between education level and stroke mortality that was observed in our study is consistent with a recent review in which the majority of studies, if not all, showed similar results [4]. The mechanisms by which SES affects stroke mortality are not well understood. Differential exposure to behavioral risk factors, particularly smoking and obesity, have been shown to play an important mediating role in the social inequality that exists in cardiovascular disease [6, 18, 19]. However, some studies have shown that adjusting for several risk factors did not explain the entire association between stroke and SES [20, 21]. Our study suggests that this differential is dependent, at least in part, on the care-pathway. Other studies have proposed this theory, but it remains controversial [4, 7–10, 22]. In the USA, patients with relatively high SES were more likely to receive post-acute stroke rehabilitation [4], while in the UK, patients with stroke who were from more deprived areas were less likely to receive a brain scan on the same day of admission [4]. A recent study in China reported that 2,283 patients showed a reduced level of antithrombotic therapy after stroke for disadvantaged people [4]. In a nation-wide study on 14,545 patients in Denmark, low-income stroke patients were less likely to receive an integrated pattern of care compared with high-income patients [9].

In the present study, we tested the role of the care-pathway in the association between education level and survival after stroke. To measure the various care-pathways, we considered data from the literature [7–9, 11, 13–16]. A wealth of evidence exists demonstrating the efficacy of early treatment in specialist care units at the hospital level (Stroke Unit), in which a multidisciplinary team cooperates to reduce the impact of neurological deficits and systemic metabolic impairment of patients who suffer an acute stroke [11]. Moreover, it has been shown that facilities with a large amount of experience and high patient volumes are associated health outcomes [7, 16]. In contrast, the optimal pharmacological strategies for acute ischemic stroke remain under debate. In the majority of cases, ischemic stroke is caused by a blood clot blocking an artery in the brain. Thus, anticoagulant drugs may have a beneficial effect. The NICE guidelines recommend the use of both anticoagulant drugs and antiplatelet agents [11]. However, the risk of bleeding is a major concern, and the routine use of any of the currently available anticoagulants remains controversial [23]. The use of antihypertensive drugs and statins has been shown to have beneficial effects; however, the evidence is not conclusive [24, 25]. Both early and post-acute rehabilitation methods are considered effective in improving long-term outcomes [11].

Our study confirms the relevant role of high-performance hospital care in the acute phase of stroke treatment and adds to the knowledge base regarding the effectiveness of pharmacological treatment in the post-acute phase. Specifically, we used an integrated approach to measure different categories of recommended drugs and found that a higher number of drugs was associated with a stronger effect on survival. We did not observe any evidence of an association between rehabilitation and survival. In this respect, it is notable that the information on rehabilitation treatment in our study was limited because we had no data on in-hospital treatments or on the use of private supplies, meaning that misclassification may have occurred. Moreover, we did not have data on health outcomes other than mortality, e.g., quality of life or functional recovery, that are considered to be more specific outcomes of rehabilitation treatment.

Despite evidence from all over the world of disparities in survival according to SES, the identification of effective strategies to tackle SES discrepancies in health care is a relevant challenge that may vary according to geographical and cultural context. [26, 27] No unique effective health policy has been proposed in Europe because it depends on the different health care organization in each country. In Italy, besides universal coverage of health services, evidence exists of the disparity in health across SES groups [28]. However, no systematic program has been implemented at the national or regional level to reduce disparities in health. Previously, the publication of data from a systematic analysis of quality indicators lead to a reduction in the SES differential in mortality in elderly patients with hip fracture [29]. A relevant result from the present study is the reduction in the SES differential in survival when patients are “exposed” to the optimal pathway of care in comparison to low-quality pathways of care. This suggests that implementing strategies to promote high quality of care in the overall population may lead to an improvement in health for more vulnerable people. The residual level of differences in survival observed in patients “exposed” to the best care-pathways may be attributed to individual clinical, behavioral or contextual risk factors.

We also evaluated the pattern of association according to sex and age. The impact of care-pathway quality on the association between education level and 1-year survival was the same for men and women, confirming previous findings [30]. For younger patients, we observed almost the same socioeconomic differential in mortality according to different scenarios of care-pathways, while in older patients, the social inequality was higher among those who received the worst care-pathway. This can be interpreted as a greater level of susceptibility to a low level of care among disadvantaged old people because of their higher prevalence of multiple concomitant diseases and unfavorable behavioral and situational risk factors.

The strengths of our study include the population-based study design, the large number of patients, the use of an integrated measure of care retrieved from different sources, the multilevel statistical approach, and the measurement of a synthetic value of probability to survive using information from both the acute and post-acute phases. Some limitations should also be recognized. First, the level of education was obtained from the discharge documents, and we did not have information on its accuracy. However, in a previous study in our region, the validity of this measure was evaluated, and they estimated that it was good [31]. Second, drug use data from our health data warehouse refer to the prescribed agents, but the actual levels of intake cannot be evaluated, as in all studies that use this information [32]. Third, the potential limitation of the statistical approach was considered; to test the robustness of the results, we performed the analysis using different statistical methods, and the results were sufficiently confirmed.

## Conclusions

This study demonstrated the real-life effectiveness of evidence-based interventions for stroke patients, highlighting the persistent differences in survival across SES groups even when taking into account quality of care. However, the findings emphasize the importance of improving the quality of a care as a tool to reduce health disparities.

### Ethics

This study was carried out in full compliance with the current privacy laws. The Department of Epidemiology is legitimised by the Lazio Regional Committee in managing and analysing data from the regional health information systems for epidemiological purposes.

### Availability of data and materials

The data used for the study are no openly available. The study was conducted with the permission of the Department of Epidemiology of Lazio Regional Health Service, the regional referral centre of epidemiological research who has full access to anonymized health information systems. The Department of Epidemiology has been authorized by Ministry of Health to use the data.

## Abbreviations

BCI: bootstrap confidence intervals
HR: hazard ratio
ICD-9-CM: international classification of diseases -9th revision-clinical modification
IPW: inverse probability weighting
OR: odds ratio
PR: probability ratio
SES: socio-economic status
